# Gibraltar Epidemic

**Published:** 1831-02

**Authors:** D. Barry

**Affiliations:** Physician to the Forces. 26, Welbeck street


THE LONDON

### Medical and Physical Journal.

No. 384, VOL. LXV[illegible].] FEBRUARY 1831. [No. 56, *New Series.*

**For many fortunate discoveries in medicine, and for the detection of numerous errors, the world is indebted to the rapid circulation of Monthly Journals; and there never existed any work to which the Faculty, in Europe and America, were under deeper obligations than to the *Medical and Physical Journal of London*, now forming a long but invaluable series.—Rush.**

### ORIGINAL PAPERS, AND CASES,

### OBTAINED FROM PUBLIC INSTITUTIONS AND OTHER AUTHENTIC SOURCES.

### GIBRALTAR EPIDEMIC.

*To* Sir Henry Halford, Bart. *President of the Royal College of Physicians, &c. &c.*

Sir: Having recently arrived from Gibraltar, I take the liberty of enclosing to you, as the head of British medical science, a few short notes on the epidemic which prevailed in that fortress, during the summer and autumn of 1828.

A residence of fourteen months on the spot, part of the time as principal medical officer; domiciliary examination of several hundred persons, either actually labouring under, or who had lately suffered from, the disease; my connexion with the various commissions of inquiry into its nature, origin, and peculiarities, brought, as may be supposed, a considerable number of facts connected with it under my notice.

In the present imperfect little sketch of so vast a subject, I have endeavoured, as much as possible, to avoid giving the impressions which these facts have left upon my mind, instead of a detail of the facts themselves. Such as it is, however, it is offered as a small contribution to the general stock of medical observation, in humble imitation of the bright example which you continue to set to the profession, by directing so much of your valuable time to interesting research, and instructive communication.

I have the honour to be, &c.

D. BARRY, M.D.
Physician to the Forces.

26, *Welbeck street; 15th February*, 1830.

Reel: 81-36-3

### *On the Gibraltar Epidemic of* 1828; by Dr. David Barry, Physician to the Forces, &c. &c.

(Read at the College of Physicians, on the 8th March, 1830.)

After the most laborious and minute inquiries, both by commissions and individuals, it was ascertained, and it is now allowed by all, that the late epidemic disease of Gibraltar first showed itself in district No. 24, one of the highest of the town.

Two children, named Fennic, brother and sister, natives of the rock, aged thirteen and ten, are returned in the burial register of the Spanish church of Gibraltar, as having died of *fever;* one on the 17th, the other on the 20th, of August; each, as was afterwards proved by evidence, on the fourth day of their respective illness: one after having vomited a liquid resembling coffee-grounds, both with yellow and livid skin.

These are the very first cases of fever that occurred on shore, from the 14th of the preceding April, of which there is even a tradition discoverable. The next case, which was fortunately detailed in writing at the time, is that of the playmate and neighbour of these children. It is as follows, copied verbatim; the first part of it from an official report made by the medical inspector of the district, to the then principal medical officer, dated 29th August; the second part from the case-book of the civil hospital, where it was entered at the moment, and where it is still to be found.

"Case. August 19, 1828. F. Cafiero, aged ten, residing on the road to Flat Bastion. I found, on visiting this boy, that he had been very low-spirited and inactive for several days, but that on Saturday he was so unwell as to keep his bed. I saw him on the 19th (of August, 1828,) at half-past four in the evening. On that day, the symptoms were a marked yellowness of the skin and eyes; pains in the forehead and region of the stomach; tongue covered with a deep fur; constant vomiting of a dark matter; bowels constipated."

(Signed)

"G. Woods, Hospital Assitant."

The following is the sequel of this case:

"Cafiero, ætat. ten; *febris remittens*; admitted 21st August, 1828, ten o'clock: just admitted. Appears to have fever of a bilious type. Has been attended by Mr. Woods, who reports the boy to have been delirious, and to have had vomiting of a dark fluid, with alvine evacuations of the same. At present, he is cool and without fever; the senses are distinct, but he looks wild; there is slight epigastric pain; skin tinged; tongue clean and moist, not loaded. To be watched. Has had calomel and Pulv. Ant., a blister to the neck, with other measures.

"22d August. No bad symptoms.

"23d. No complaint.

"24th. Dismissed.

"A true copy, from the case-book of the civil hospital, detailed by Mr. FRASER."

(Signed)

"JOHN MILLAR, Assist. Staff Surgeon."

These, and many other documents of a similar nature, and of that time, are the more valuable from having been written "*ante litem motam*," and because they furnish authentic, unprejudiced records of the very earliest cases, which, when compared with those of the middle and latter periods of the epidemic, will show, that the disease commenced at once, with its characteristic signs, and agreeably to the precept of Horace, as to tragic perfection, "Servetur ad imum qualis ab incepto processerat, et sibi constet."

The first case of the disease, which occurred amongst the military part of the population, is the following, abridged from the hospital register of the 12th Foot:

"CASE, dated evening, 2d September, 1828.

First day. "Sergeant H., taken ill this morning, with cold shivering, pain in the epigastrium, severe headach, prostration of strength, nausea, and retchings; pulse quick and full; skin hot, eyes turgid, countenance anxious, bowels costive.

Second day. "A restless night; pulse still full, skin hot and dry, tongue whitish; blood drawn shows a yellow crust.—Evening. Tossing about; irritability of stomach; tongue loaded, thirst moderate; dejections dark and watery.

Third day. "Constant tossing from side to side; a yellow tinge about the eyes and chest; no headach; pulse 100.—Evening. Vomiting a dark liquid, not unlike dark olive grounds, which precipitate to the bottom of the basin; pulse feeble and irregular; extremities cold.

Fourth day. "Vomiting of darkish fluid; delirium; incessant tossing; pulse scarcely perceptible and sinking. Death at nine o'clock this morning."

This man lodged with his reputed wife, in Flat Bastion road, above Fennic's, and nearly opposite to Cafiero's house, in 24 district.

The following note refers to one of the last cases:

"14th December, 1828. Private D., of the 42d Royal Highlanders. Fourth day of the disease: he is yellow; bleeds from the nose and gums; tongue loaded; skin cool; great prostration; vomits a liquid, precisely resembling ill-prepared coffee.

Fifth day, eight o'clock P.M. "Appears much better: has not vomited for the last two hours.

Sixth day. "Vomiting black liquid; delirium; hiccup; tremulous pulse; suppression of urine. Death."

Mild cases, of which there were a great number, were all, more or less, of the following kind:

Sensation of cold, frontal headach, white tongue, slight nausea, flushed face, injected eye, hot skin, costive bowels, sweat, within the first twenty-four hours; sweat continued, refreshing sleep, within the second or third twenty-four hours. Convalescence.

A yellow tinge of eye, or skin, often, though not always, marked even the mildest case, towards its close. This was observable in very young children, who generally had the disease in its very gentlest form.

Five thousand five hundred and forty-three cases, from August to Christmas, of which 1631 were fatal, could such a number have been recorded, would echo, and the hundreds of them that have been recorded do echo, one or other of those I have just described. They would prove that this disease, reduced to its most elementary form of expression, consisted of one, and only one, febrile paroxysm, terminating, from the second to the sixth day, either in a rapid and permanent return to health, or in the precursory, but apyrectic, signs of speedy dissolution,—black vomit, hiccup, and passive hemorrhagies.

The most constant appearances after death were yellowness of the skin, generally with livid patches, not effects of position; a black, japanned appearance, shading off into red, over some inches of the cardiac lining of the œsophagus; black, brown liquid in the stomach, sometimes amounting to two or three pounds, though vomiting had continued to the last;* dark red patches, as if formed by ecchymosis from a bruise, often with honeycomb corrugations of the inner membrane, the red patches extending into the duodenum; pitchy, black matter in the small, or large intestines, or both. The liver, in all the cases that I saw examined, looked as if it had been partially boiled: that is, large portions of its surface were of a bright fawn colour, whilst the intermediate portions preserved the natural hue, and seemed as if they had not been immersed in the boiling liquid. Mr. Fraser, of the civil hos-

* Often when none had ever been vomited.

pital, notes this appearance in his dissection report of the washerwoman, Mary Silcox, on the 1st September; Mr. Amiel, in the case of Sergeant H., just quoted; besides a host of others. Ulceration of the mucous lining of the intestines, or elevation of Peyer's glands, was found but once in this epidemic, by that eminent pathologist, M. Louis; and in this case the symptoms were those of protracted typhus. All the other organs were usually found healthy, or varying in a thousand inconstant appearances, from which nothing could be determined.

But what first produced this disease, in Gibraltar, and how was it propagated?

After an investigation which occupied forty-seven long sittings of a Board, of which I had the honour of being a member and secretary, it appeared to have been satisfactorily proved, that the spring and summer of 1828, had been mild, healthy, and rather cooler, than those of 1827; that no atmospheric, nor other phenomena, had been noticed in the territory, or its neighbourhood, different from those which had occurred in any of the five preceding years; that the size, comfort, and ventilation, of the dwellings, of all classes, had been increased; handsome public and private buildings erected; spacious squares, gardens, and promenades, constructed; the supply of wholesome water from new tanks and wells, of fresh meat, vegetables, and fruit; the cleanliness of the streets, and the construction of drains, had been progressively improving, for more than ten years, and were, at the breaking out of the epidemic, in a high state of perfection; that the population of the whole territory, military, civil, permanent, and floating, had never exceeded 21,000, since the year 1826, scattered over a surface of nearly three miles long, by a quarter broad, the bay, the neutral ground, and the village of Catalan bay; that crime was almost unknown, and that there was not a beggar on the rock.

These facts repose on documents as incontrovertible, and on the testimony of men as honourable, as any in existence. We cannot, then, assume an opposite state of things, within the fortress, as a source of disease, nor look to that just detailed, for a producing cause.

Emanations from drains, sewers, &c., were accused so early as the 29th August, and those who so accused them asserted, on the 11th September, that the disease was already checked by the ablution of these drains. But a glance at the plan will show, at once, that district No. 24, where, as we have seen, the disease first broke out, and to which it was confined for three weeks, must, from its position, have the fewest, and the smallest drains, and that these must be the cleanest, from their great declivity, and from their commencing on the spot.

So much for possible sources of disease within the fortress: now for those without.

During the summer of 1828, several vessels had arrived from the West Indies, and various ports of South America; on board of which as many as ten persons had died, on their passage to Gibraltar. On board of one ship, which arrived from the Havannah, on the 27th June, and was admitted to pratique on the 6th August, two persons had died, and nine had been ill, on her passage; but it could not be satisfactorily ascertained of what description of fever, for it was allowed to have been of *some fever.* Three others were sick in this vessel, whilst she lay in quarantine. One of the latter was admitted into the civil hospital, on the 7th August, where his name is to be found entered thus by Mr. Fraser:

"8th August, John Barrow, aged thirty; *febris intermittens;* admitted yesterday, from the Havannah, where he had fever." This man was discharged on the 10th, without any further notice, and without his having had, as it now appears, any paroxysm of ague.

It was given in evidence by the boy Cafiero, that he and the Fennic children, with their father, had been on board *some ship,* on the Sunday before the first of them fell ill, and staid on board, above an hour. They went and returned in the father's bumboat.

Could the seminium of the disease have been found in any of the vessels alluded to, and thence brought on shore?

Be the answer to this question what it may, it is certain, that, besides the Fennic children, their father, who died on the 6th September, and the boy Cafiero, three other persons were attacked in Fennic's house, before the end of August; that the sister of the health-guard, who landed from the Havannah ship on the 6th, with his effects, washed his clothes on the 11th, and fell sick of fever on the 21st August; that the immediate neighbourhood of these persons, in district 24, and the civil hospital, were exclusively infected, up to the 2d September, to which time, from the death of the first Fennic child, fifty persons had been attacked, and six died, besides three other deaths, that were not satisfactorily accounted for.

The disease broke out at a moment of the most perfect public health, which had been but little interrupted for thirteen years. It first seized the Fennic family, and others, who either actually were, or might be fairly presumed to have been, most connected with ships and their impurities; such as the families of health-guards, washer-women, bumboatmen, and petty smugglers. It was confined to such persons, or their immediate neighbours and connexions, for more than three weeks, in one of the most thinly inhabited districts of the town, where washer-women resorted, on account of the space it afforded for drying ground. When it had once entered, it never quitted a family, or a house, until it had attacked every individual that *had not* passed it in some former year, invariably sparing those who *had.* When a person, labouring under this disease, was removed to a house previously healthy, not only that house, but the circle of houses immediately around it, soon became infected. Where perfect isolation was observed, the disease did not enter.

The soldier servants of the military hospitals were found to fall so fast, that civil attendants became necessary. Sixty-one of these were employed, of whom two only had *not* passed the disease in some former epidemic.

Of 164 military attendants on the sick, 141 were attacked. Of the sixty-one civil attendants, two only were attacked, and these were the two who had not had the disease before.*

I shall abstain from arranging this disease under any nosological name. It would, perhaps, have been better for science, certainly for humanity, if it had never received one. When a single paroxysm, of a few short hours, terminates the attack for ever; when the signs and the characters that I have enumerated are observed; when the march of infection and death appears to be from fixed points; it were a pity, and ought perhaps to be considered a crime, to allow a theory, which a nosographical name may imply, to suspend, even for a moment, our efforts to stop that march.

Our knowledge of the solitary fact, that this disease never attacks the same individual more than once, is ten thousand times more valuable than all that we know of it besides. It has already enabled Dr. Pym, to whom mankind is mainly indebted for having placed this fact beyond the pale of doubt, to cut short, at once, an incipient epidemic in Gibraltar, in 1810. He separated the first sick and the suspected from the healthy population, using as his instruments of separation those who had acquired the necessary immunity in some former epidemic year. This proceeding now forms the basis of a sanatary law in Spain, and has already been success-

* Taken from the official returns of the regimental surgeons of the seven corps composing the garrison.

By a census taken in the spring of 1829, it appeared that above 6,000 persons had passed the disease previously to 1828, not one of whom was attacked in the latter year.—D. B.

fully repeated in Barbadoes by Mr. Green, inspector of hospitals, in 1821.

*Treatment.* Three leading plans strike our attention in the therapeutic records of the Gibraltar epidemic.

1st. The mercurial plan, with a view to affect the mouth.

The 2d. Early, copious, and repeated bleeding.

The 3d. Mild, frequent, oily purgatives, with acid diluents.

From a comparative analysis of all the returns, the first of these modes of treatment appears to have been the least successful; the third the most successful. But, to arrive at more precise conclusions on this point, the class, age, sex of the patient, with other important elements, should be taken into calculation, and this would lead to details inadmissible on the present occasion.

During the autumn of 1829, there were more common agues, malignant tertians, and remittent fevers, with distinct paroxysms, in Andalusia, in the immediate neighbourhood of Gibraltar, than had been known there for twenty years: yet all within the fortress remained perfectly exempt from these fevers. Seven cases, with yellow skin, from May to September, attracted notice in the British territory: three had no fever; four had passed the epidemic disease, either in 1804, 1813, or 1828; two proved fatal;* but neither the signs during life, nor the characters after death, which I have enumerated, were found in any of them.

* In all, the urine was highly icteric, and dyed linen yellow.

